# ^31^P multi‐echo MRSI with low B_1_^+^ dual‐band refocusing RF pulses

**DOI:** 10.1002/nbm.5273

**Published:** 2024-10-10

**Authors:** Zahra Shams, Wybe J. M. van der Kemp, Dennis W. J. Klomp, Evita C. Wiegers, Jannie P. Wijnen

**Affiliations:** ^1^ Center for Image Sciences University Medical Center Utrecht Utrecht The Netherlands

**Keywords:** dual‐band refocusing pulse, increased SNR per unit of time, low‐power RF pulse, phosphorus multi‐echo MRSI, PME and PDE detection, ultra‐high field MRSI

## Abstract

^31^P magnetic resonance spectroscopy (MRS) can spectrally resolve metabolites involved in phospholipid metabolism whose levels are altered in many cancers. Ultra‐high field facilitates the detection of phosphomonoesters (PMEs) and phosphodiesters (PDEs) with increased SNR and spectral resolution. Utilizing multi‐echo MR spectroscopic imaging (MRSI) further enhances SNR and enables T_2_ information estimation per metabolite.

To address the specific absorption rate (SAR) challenges associated with high‐power demanding adiabatic or composite block pulses in multi‐echo phosphorus imaging, we present a dual‐band refocusing RF pulse designed for operation at B_1_ amplitudes of 14.8 μT which holds potential for integration into multi‐echo sequences.

Phantom and in vivo experiments conducted in the brain at 7 Tesla validated the effectiveness of this low‐power dual‐band RF pulse. Furthermore, we implemented the dual‐band RF pulse into a multi‐echo MRSI sequence where it offered the potential to increase the number of echo pulses within the same acquisition time compared to high‐power adiabatic implementation, demonstrating its feasibility and practicality.

## Graphical abstract

In this study, considering the benefits of ultra‐high field ^31^P MRS for cancer‐related metabolite analysis, we introduced a low‐power dual‐band RF pulse to address SAR challenges in multi‐echo phosphorus imaging, demonstrating its effectiveness for detection of phosphomonoester (PME) and phosphodiester (PDE) in both phantom and in‐vivo brain experiments at 7 T. Incorporating this dual‐band RF pulse into a multi‐echo MRS imaging sequence offers a promising avenue for extending its application to whole‐body imaging under conditions of low B_1_ field strength.

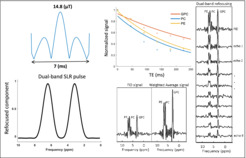

**List of Abbreviations**
AMESING: Adiabatic Multi‐Echo Spectroscopic ImagiNG
CPMG: Carr‐Purcell‐Meiboom‐Gill
ETL: Echo Train Length
FA: Flip Angle
FID: Free Induction Decay
FSE: Fast Spin Echo
FWHM: Full Width at Half Maximum
GPC: Glycerol PhosphorylCholine
GPE: Glycerol PhosphorylEthanolamine
MRS: Magnetic Resonance Spectroscopy
MRSI: MR Spectroscopic Imaging
NOE: Nuclear Overhauser Enhancement
NAV: Number of AVerages
PA: Pulse Acquire
PME: PhosphoMonoEster
PC: PhosphorylCholine
PCr: PhosphoCreatine
PDE: PhosphoDiEster
PE: PhosphorylEthanolamine
P_i_
: inorganic Phosphate
ppm: Parts Per Million
PRESS: Point RESolved Spectroscopy
SAR: Specific Absorption Rate
SE: Spin Echo
SLR: Shinnar‐LeRoux
SNR: Signal‐to‐Noise Ratio
tCho: total Choline
TE: Echo Time
TR: Repetition Time

## 1 INTRODUCTION

Alterations of phospholipid metabolism have been recognized as a hallmark of cancer.^1^, ^2^ One of the biomarkers that characterize phospholipid metabolism is the total choline (tCho) content of the tissue. Cancer or therapy‐related changes in tCho levels can be monitored by in vivo ^1^H‐MRS. However, in vivo ^1^H Magnetic Resonance Spectroscopy (MRS) cannot distinguish between phosphorylated compounds in phospholipid metabolism such as phosphomonoesters (PMEs; phosphorylcholine (PC), phosphorylethanolamine (PE)) and phosphodiesters (PDEs; glycerol phosphorylcholine (GPC), glycerol phosphorylethanolamine (GPE)), as the chemical shift dispersion is too small. PDE and PME signals can be resolved in vivo by using ^31^P MRS. The increased ratio of PME over PDE (PC to GPC and PE to GPE ratios) has been related to tumor growth and aggressiveness in many cancers^2^, ^3^ while a decrease in this ratio during therapy has been indicative of successful treatment of e.g. breast tumors.^4^, ^5^, ^6^, ^7^

^31^P MRS data can be obtained at 1.5 or 3 Tesla. However, the use of ^31^P MRS is hampered due to the low gyromagnetic ratio and low abundance in human body tissue which leads to lower NMR sensitivity of ^31^P MRS when compared to ^1^H MRS. Higher magnetic field strengths, such as 7 Tesla, can be used to increase the signal‐to‐noise ratio (SNR). The ^31^P MR sensitivity can also be improved by proton decoupling which is achieved by the application of high‐power broadband RF pulses,^8^ yet high specific absorption rates (SAR) impede the use of this technique at ultrahigh magnetic field.^9^, ^10^ ^31^P‐^1^H nuclear Overhauser enhancement (NOE) is another approach for phosphorous signal enhancement that is performed by applying low‐power proton irradiation to saturate the proton in a heteronuclear two‐spin‐system, during the phosphorous signal acquisition. Because of the low‐power pulse, NOE is in theory more suitable than proton decoupling for use in ultra‐high field.^11^ However, the signal enhancement by NOE (per metabolite or across the experiments) depends on the T_1_, which can introduce bias when studying pathologies where T_1_ may be altered.^10^, ^12^, ^13^ Previous studies have shown that the most reproducible results (i.e. smaller fit errors) and consistent NOE enhancement were observed for the larger peaks in the ^31^P spectrum, such as phosphocreatine (PCr).^10^, ^14^, ^15^ NOE at 7 T imposes higher SAR with minimal gain in SNR. Polarization transfer methods have also been used to increase SNR of ^31^P signals.^16^, ^17^, ^18^ For example, the BINEPT (B_1_‐insensitive nuclear enhancement through polarization transfer) technique using adiabatic pulses could resolve low levels of PMEs and PDEs in non‐tumorous breast tissue within a clinically acceptable acquisition time.^18^ However, these techniques experience signal degradation due to decreased T_2_* values under poor B_0_ shimming conditions. Line broadening frequently occurs in tissues with pronounced susceptibility interfaces. Higher SNR and insensitivity to B_0_ shimming can be achieved by utilizing multi‐echo approaches such as the adiabatic multi‐echo spectroscopic imaging (AMESING) sequence. Additional advantages of using this technique for phosphorous imaging are the T_2_‐weighted SNR enhancement, for an increased metabolite sensitivity, and the access to T_2_ information per metabolite.^19^, ^20^ Yet, this technique with the use of adiabatic pulses is of interest, particularly in the body where B_0_ and B_1_ homogeneity is poor. The adiabatic pulses in the AMESING implementation work at a significant amount of power to meet the bandwidth requirement (B_1_^+^ ~ 100 μT) which can only be achieved by surface coils.

Using volume coils instead of surface coils enables the application of non‐adiabatic RF pulses at more uniform and lower B_1_^+^ fields. Previously, a multi‐echo 3D MRSI with composite block pulses for refocusing was implemented to determine the T_2_ relaxation times of the inorganic phosphate (Pi), PDEs, and PMEs in the human brain at 7 T using a dual‐tuned ^31^P‐^1^H quadrature head coil.^21^ The composite block pulse for this purpose refocused the spins within a 4‐ppm frequency range of interest (2.9 ppm–6.9 ppm) at a B_1_^+^ field of ~ 40 μT. At lower B_1_^+^ fields and for a larger field of views in the body, multi‐echo MRSI was investigated using the same composite block pulses requiring a B_1_^+^ of ~ 15 μT, with a consequently narrower frequency range of ~2 ppm targeting only one metabolite (e.g. inorganic phosphate (P_i_) or phosphocreatine (PCr)) or one group of metabolites (e.g. only PDE, or only PME).^22^

This research aims to develop an efficient pulse sequence, using a dual‐band refocusing pulse, at the B_1_^+^ restriction of 15 μT, with the goal of boosting sensitivity for detecting metabolite signals of PDE and PME. Increased sensitivity can be achieved through the utilization of a high number of echoes, thereby enhancing the signal‐to‐noise ratio (SNR) per unit of time. Phantom and in vivo experiments in the brain are shown as proof of successful operation for this dual‐band pulse at 14.8 μT.

## 2 METHODS

### 2.1 Dual‐band refocusing pulse generation

A Shinnar‐LeRoux (SLR)‐optimized dual‐band refocusing RF pulse was designed using the MATPULSE tool in MATLAB (mathworks, R2021a), which provides computer‐optimized shaped (amplitude‐modulated) RF pulses.^23^ The bandwidth of the two refocusing bands of the dual‐band RF pulse was optimized such that the B_1_ amplitude did not exceed 15 μT within a reasonable pulse time duration (i.e., < 8 ms). The pulse tip angle and duration were specified to 180° and 7 ms with 128 pulse steps. Other pulse characteristics were as follows: the transition band (full‐width half maximum [FWHM] of each of the refocusing bands) was 0.17 kHz estimated by setting the passband and rejection ripples to 30% and 0.05%, respectively; The separation width (peak‐to‐peak distance minus the transition band) was 0.24 kHz.

Additionally, the refocusing profiles of two other pulses were simulated to compare the required B_1_^+^ amplitudes and relative SAR levels with those of the dual‐band pulse. One of these pulses was a single‐band SLR RF pulse produced through Bloch simulations using the MATPULSE tool. The other pulse involved amplitude and frequency modulation extracted from the Philips pulse archive, specifically employed in the Point RESolved Spectroscopy (PRESS) sequence (referred to as Refoman 6).

The relative SAR of the RF pulses was calculated according to:

(1) SAR∝B1,rms,pulse+2=1Tp∫0TpB1,pulse+t2dt

where $T_{p}$ is the duration of the RF pulse and $B_{1}^{+}\left(t\right)$ is the pulse shape as a function of time (t).

### 2.2 Simulations

In MR imaging, a widely incorporated method to increase SNR per unit of time is the use of three‐dimensional fast spin echo (FSE) with modulated refocusing flip angles (FA).^24^, ^25^ This employs a very long echo train length (ETL) using variable, low FAs. We simulated the use of variable FAs FSE in the ^31^P regime as a new approach for gaining SNR per unit of time within specific absorption rate (SAR) limits. The results were compared with a multi‐echo technique with full refocusing pulses of 180° (see Supplementary Materials).

To gain insights into how B_0_ inhomogeneity affects the accuracy of the dual‐band refocusing pulse, the effect of varying the offset frequency was simulated. Simulations were conducted using TopSpin 4.0.9 (Bruker, Billerica, MA, USA) to evaluate the impact of B_0_ inhomogeneity on the refocusing of PDE and PME signals. These simulations utilized a spin echo sequence with a block excitation followed by the dual‐band echo pulse. The simulated metabolites included PE, PC, GPC, GPE, and P_i_, and we applied offset frequencies to the pulse in 0.1 ppm increments, ranging from −1 ppm to 1 ppm.

### 2.3 Data acquisition

MR measurements were carried out on a 7 Tesla MR system (Philips, Achieva, Best, NL). In vitro measurements were acquired on a sphere phantom (diameter 9 cm) enclosing two other small spheres (diameter 4 cm) using a homebuilt dual‐tuned ^31^P‐^1^H head coil. One of the spheres contained PMEs (i.e., PC and PE) and PDE (i.e., GPC), in similar concentrations (50mM); and the other sphere contained 200mM Pi. In addition, measurements were performed in the brains of one healthy volunteer (female, 28 years). The in vivo study was approved by the UMC Utrecht Medical Ethical Committee and written informed consent was obtained from the participant.

### 2.4 Validation of the dual‐band refocusing pulse shape

To verify the refocusing profile of the dual‐band pulse, we performed spin echo (SE) experiments with the dual‐band pulse as a refocusing pulse; sequence parameters were TE/TR 9.98 (ms)/6000 (ms), spectral width 6400 Hz, samples 512, number of averages (NAV) 10, phase cycle 1, no volume localization and with a block pulse of 90° for signal excitation. To evaluate the performance of the dual‐band pulse, we compared the signal intensities of PDE and PME qualitatively to those from a pulse acquire (PA) sequence with parameters: TE/TR 0.2 (ms) /1000 (ms), block excitation with a flip angle of 45°, spectral width 5000 Hz, samples 512, NAV 4, phase cycles 2. These experiments were performed both in the phantom and in vivo.

### 2.5 Implementation of the multi‐echo sequence

The scan protocols for both spin echo and multi‐echo experiments (either in a phantom or in vivo) consisted of a T_1_w scan, a B_0_ map for image‐based shimming based on FASTMAP^26^ and a ^31^P flip angle calibration scan using pulse‐acquire with asymmetric sinc pulses with 12 steps of 20 degrees in the nominal range of 40–280 degrees. The multi‐echo AMESING sequence^19^ was modified such that the excitation was performed using a rectangular 90° pulse, succeeded by 180° Shinnar‐LeRoux dual‐band refocusing pulses (7 ms) at B_1_^+^ 14.8 μT. A single free induction decay (FID) was acquired by means of a pulse‐acquire (block pulse) and 5 (Phantom and in vivo), 9 (phantom), and 15 (phantom) full echoes in one k‐space step while k‐space data were sampled spherically. As a reference, we performed the same experiment of one FID with five consecutive echoes on a phantom using the sequence with adiabatic pulses at a B_1_^+^ of approximately 100 μT. This was achievable only with the phantom using a homebuilt dual‐tuned surface coil set‐up.^9^, ^19^ An adiabatic half passage pulse was used for excitation and BIR‐4180° pulses for refocusing.^19^ All multi‐echo MRSI experiments were performed with a spherically sampled k‐space matrix size of 8 × 8 × 8, isotropic spatial resolution of 20 mm, NAV 1, spectral data points 256, spectral width 8200 Hz, ΔTE 45 ms, TR 5 s (in vivo), and 4 s (phantom). The total scan times were 25 min (in vivo) and 17 min (phantom).

### 2.6 Data processing

The FID and echoes were zero‐filled to 1024 points in the time domain. The FID signal was first‐order phased for the acquisition delay, and the echo signals were corrected for the zero‐order phase following the Fourier transform. Spectral fitting was conducted using the AMARES algorithm within jMRUI.^27^, ^28^ Following spectral fitting, the amplitudes of each metabolite in the FID and echoes were fitted to an exponential decay function to obtain T_2_ values. To compare the SNR levels among the spectra with different numbers of symmetric echoes, the weighted average SNR (${\mathrm{SNR}}_{\mathrm{wa}}$), as previously described,^19^ was calculated as in Equation 2.

(2) $$S_{\mathrm{wa}}=S0\frac{1+2\sum_{i=1,..,n}\mathrm{Si}w_{i}}{1+2\sum w_{i}},\;{\mathrm{SNR}}_{\mathrm{wa}}=\frac{S_{\mathrm{wa}}}{\sigma_{\mathrm{wa}}}=\mathrm{SNR}0\frac{1+2\sum_{i=1,..,n}\mathrm{Si}w_{i}}{\sqrt{1+2\sum w_{i}^{2}}}$$

Where S0, Si, SNR0, $w_{i}$, $S_{\mathrm{wa}}$, n, and $\sigma_{\mathrm{wa}}$ represent FID signal, i ^th^ echo signal, FID SNR, signal weight which is the T_2_ decay, weighted average signal, and noise, respectively. This equation illustrates the relationship between the SNR of the combined FID signal and echo signals and the SNR of the FID signal alone.

## 3 RESULTS

**FIGURE 1:**
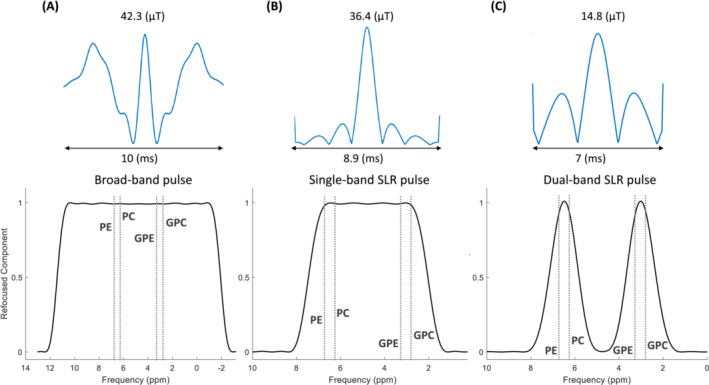
RF pulse shapes and their corresponding refocused component for (A) a broad‐band RF pulse exported from the Philips pulse archive, (B) a designed single‐band RF pulse using MATPULSE tool, and (C) the designed dual‐band refocusing RF pulse for the purpose of refocusing PDE and PME spins with a bandwidth of 0.17 kHz and separation width of 0.24 kHz. Refocused component values of the PDE and PME metabolites for the broad‐band, single‐band SLR, and dual‐band RF pulses are (PE = PC = GPC = GPE ≈ 0.99), (PE = GPC ≈ 0.98 and PC = GPE ≈ 0.99), and (PE = PC = GPC = GPE ≈ 0.95), respectively.

The profile of a broadband, the dual‐band, and a single‐band spin echo RF pulses with their corresponding refocused components can be seen in Figure 1. A broad‐band RF pulse that covers most of the ^31^P MR spectrum, including the PME and PDE resonances, requires a B_1_^+^ amplitude exceeding 40 μT and a duration of 10 ms, yet it still does not cover the entire spectrum (Figure 1A). Using a dual‐band pulse with refocusing bands placed at the PDE and PME resonances at 3.0–3.5 ppm and 6.2–6.8 ppm, respectively, requires only an amplitude of 14.8 μT and a duration of 7 ms (Figure 1B). While, if a single band refocusing pulse were employed, it would necessitate a B_1_^+^ of approximately 36 μT (with a pulse duration of 8.9 ms) to encompass the frequency range including both PDE and PME (Figure 1C). Even more power than 36.4 μT is needed when a shorter RF pulse of 7 ms instead of 8.9 ms is desired. Additionally, the relative SAR value is much lower for the dual‐band RF pulse (Table 1) compared to the single‐band and broadband RF pulse.

**TABLE 1:** Relative SAR (specific absorption rate) values for generated and simulated RF pulses are in Figure 1.

RF pulse	Dual‐band	Single‐band	Broadband
B_1max_ (μT)	14.8	36.4	42.3
T (ms)	7	8.9	10
SAR_rel_	0.12	0.33	2.45

**FIGURE 2:**
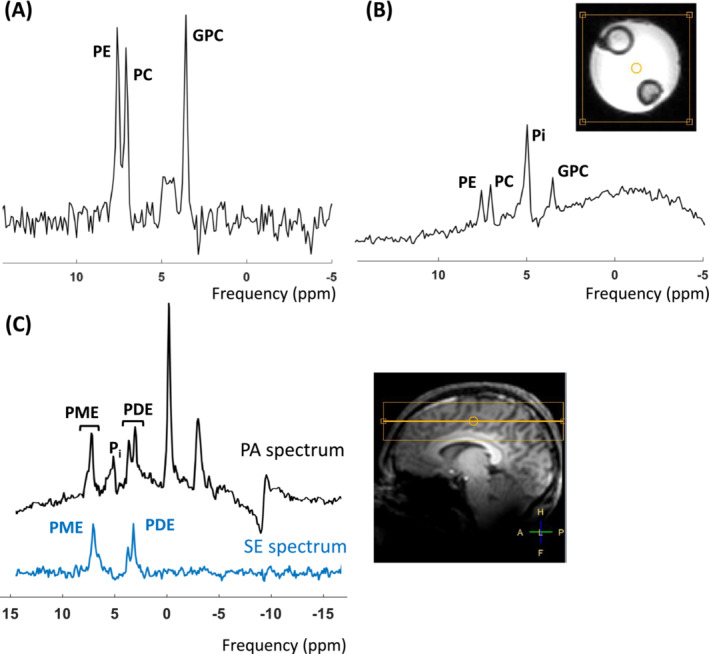
MR spectra acquired from a phantom with (A) a spin echo and (B) a pulse acquire acquisitions. (C) The MR spectra (scaled to the same noise level) acquired in vivo from a pulse acquire and a spin echo acquisition with the dual‐band RF pulse aimed to hit the PDE and PME spin groups from the entire head. All MR spectra were displayed using a 10‐Hz Lorentzian line‐broadening filter. Note the well‐refocused signals of PME and PDE in the brain. A small residual peak of (partly) refocused inorganic phosphate (P_i_) at 4.9 ppm is visible as well.

In both the phantom, containing Pi, GPC, PC, and PE compartments (presented in Figure 2A,B), as well as in the human brain (shown in Figure 2C), discernible refocused signals from the two groups of spins can be observed, resembling the signals acquired with the PA acquisition.

**FIGURE 3:**
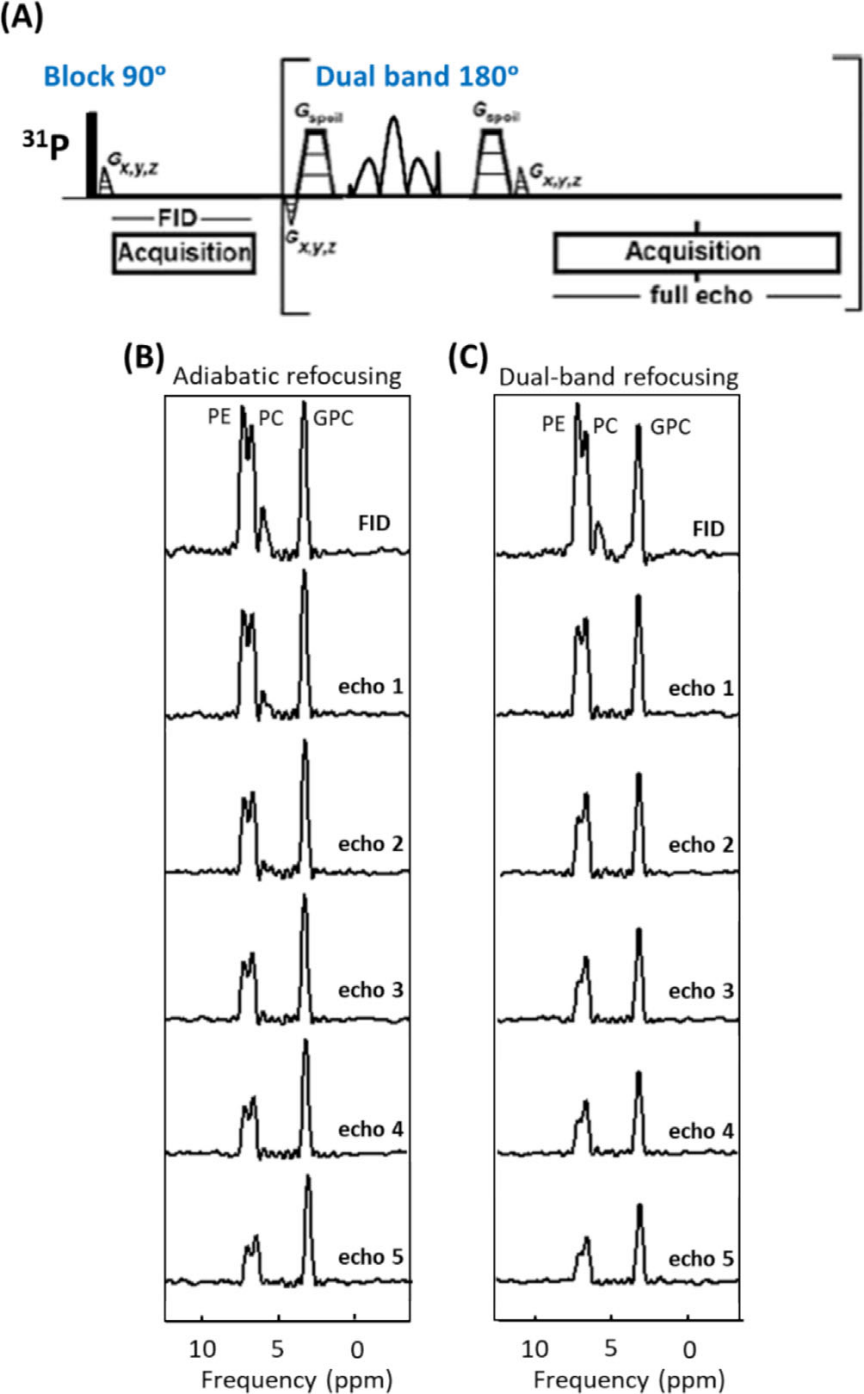
(A) Pulse sequence diagram for multi‐echo sequence with block pulse excitation and dual‐band refocusing pulses. MR spectra from a phantom containing PE, PC, and GPC employing a multi‐echo sequence with (B) adiabatic excitation and refocusing, and (C) block excitation and dual‐band refocusing pulses. Experiments corresponding to A and B were performed using a surface coil at B_1_^+^ of 100 μT and 15 μT, respectively. The voxel did not contain any P_i_.

Figure 3 shows the multi‐echo sequence diagram with block excitation and dual‐band refocusing pulses. This figure depicts the MR spectra acquired from the same phantom as displayed in Figure 2, but using the multi‐echo sequence with adiabatic excitation and refocusing (Figure 3B), and with block excitation and dual‐band refocusing (Figure 3C). All the MR spectra are from a voxel that contained the sphere with GPC, PC, and PE. The very short sampling time of the FID and echoes resulted in a truncation artifact in the MR spectra. The 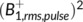 normalization in relation to the block pulse, serving as a SAR metric, resulted in a value of 0.2 for the dual‐band RF pulse and 62.2 for the adiabatic BIR4 pulses with an amplitude of approximately ~ 100 μT and a duration of 8 ms.

**FIGURE 4:**
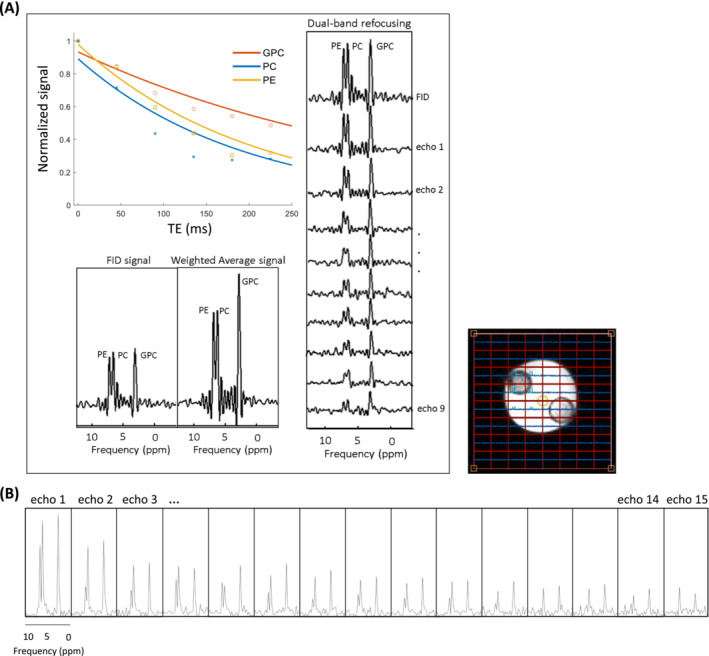
MR spectra from a phantom containing PE, PC, and GPC acquired with multi‐echo spectroscopic imaging using low‐power dual‐band RF pulses. (A) SNR increased by a factor of 2 after incorporating the weighted average (equation 2) of 9 echo signals compared to only the FID signal. T_2_ values for GPC, PC, and PE are 379 ms, 193 ms, and 204 ms respectively. (B) Increased SNR per unit of time can be achieved by using higher number of echo spectra using low‐power dual‐band RF pulses.

Figure 4A shows the MR spectra from a voxel containing GPC and PC and PE signals using the multi‐echo sequence with the implementation of 9 dual‐band RF pulses. SNR of the signal of GPC is increased by more than a factor of 2 after implementing 9 echoes compared to the FID only. The T_2_ decay curves of PC, PE, and GPC signals are plotted as a function of echo time which resulted in T_2_ values of 379 ms, 193 ms, and 204 ms for GPC, PC, and PE, respectively. The use of the low‐power dual‐band RF pulse allowed the implementation of an even greater number of echo pulses—up to 15 pulses, compared to 9—within the same TR, while still remaining within SAR limits (Figure 4B). This resulted in a 2.3‐fold increase in the SNR of the GPC signal, as calculated using Equation 2. As the number of echo pulses increases, the SNR continues to improve. However, the SNR vs. echo number curve eventually saturates, reaching a peak of approximately 2.44, indicating an optimal number of echoes. Beyond this point, adding more pulses does not further enhance the weighted average SNR (Figure S2A).

**FIGURE 5:**
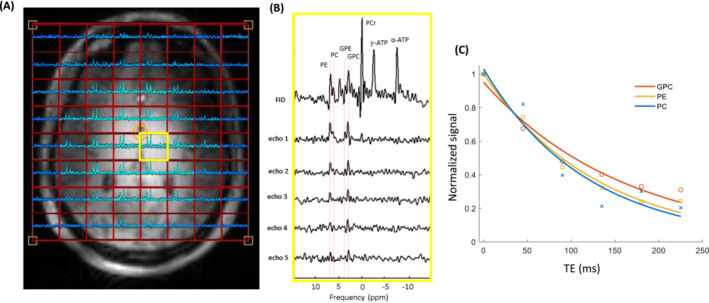
In vivo MR spectra obtained using the dual‐band multi‐echo (5 echoes) sequence. (A) Spectra from the first echo spectra captured from the central slice of the ^31^P 3D matrix. (B) FID and 5 echoes spectra acquired from the yellow voxel in the central slice of the ^31^P 3D matrix. Distinct signals of GPC, PE, and PC are resolved. (C) T_2_ decay curves for GPC, PE, and PC, exhibiting T_2_ values of 161 ms, 129 ms, and 118 ms, respectively. The experiments were conducted utilizing a quadrature dual‐tuned ^31^P‐^1^H head coil with a (^31^P) B_1_^+^ of 15 μT.

Figure 5 shows the in vivo results of dual‐band multi‐echo spectroscopic imaging in the brain. The figure presents the resolved PDE and PME signals of 5 subsequent echoes following a single FID. The resulting T_2_ values for GPC, PE, and PC in the brain were 161 ms, 129 ms, and 118 ms, respectively.

**FIGURE 6:**
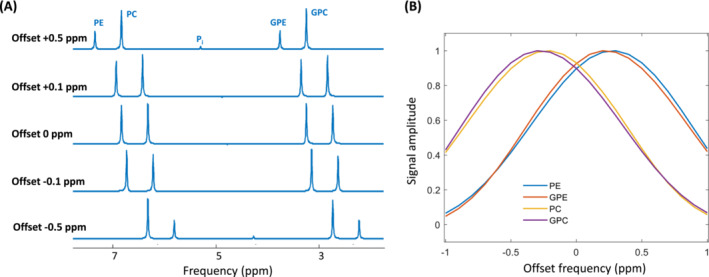
Simulation outcomes investigating the influence of B_0_ inhomogeneity on PME and PDE signal intensities. (A) Simulated spectra for five different frequency offsets. (B) Behavior of PE, PC, GPE, and GPC signal in relation to the offset frequency.

The impact of B_0_ inhomogeneity on PDE and PME spectra is illustrated in Figure 6. A slight frequency offset of 0.1 ppm (approximately 12 Hz) minimally influences the signal for all PDE and PME components. However, when a frequency offset of 0.5 ppm (around 60 Hz) is applied, there is a noticeable decrease in either PE and GPE signals or PC and GPC signals. It is important to note that this reduction in signal levels will not alter the ratio between the PDE and PME signals, as both groups are affected equally.

## 4 DISCUSSION

We introduced a dual‐band refocusing RF pulse with a low B_1_^+^ of less than 15 μT for selective refocusing of the spin groups of PMEs and PDEs; which are important metabolites in for example cancer biology. We showed ^31^P MRSI data of a phantom and the brain which verifies the inversion profile and performance of the dual‐band refocusing RF pulse. Compared to a single band RF pulse with hardly sufficient bandwidth for refocusing of PMEs and PDEs, the SAR is reduced by almost a factor of 3. Because of its low power demand, the low B_1_^+^ amplitude dual‐band RF pulse could be implemented in a multi‐echo sequence.

The focus of this study was to develop an efficient phosphorous imaging sequence to detect PDE and PME with the aim of increasing SNR per unit of time, at the B_1_^+^ limitation of 15 μT. We showed that SNR increased by a factor of 2 compared to the FID acquisition. In MR imaging, a widely incorporated method to increase SNR per unit of time is the use of three‐dimensional Fast Spin Echo (FSE) with modulated refocusing flip angles (FA).^24^, ^25^ This employs a very long echo train length (ETL; more than 100 in the brain) using variable, low FAs. Because of the relatively long relaxation time of ^31^P metabolites in comparison to ^1^H imaging, utilizing a full 180‐degree multi‐echo sequence instead of a modulated Fast FSE technique could result in improved Signal‐to‐Noise Ratio (SNR) (Figures S1 and S2). We implemented the dual‐band pulse in a full 180‐degree multi‐echo MRSI sequence and validated the approach in both a phantom and in vivo. Using the low‐power dual‐band RF pulses, the number of echo pulses could be increased compared to an implementation with adiabatic pulses (relative SAR of 0.2 compared to 62.3) within a TR of 5 s, which results in a higher SNR per time unit. This implies that the number of dual‐band echo pulses can be increased compared to the adiabatic implementation within the same TR of 5 seconds. A formal SNR comparison with other ^31^P MRSI techniques, such as NOE‐enhanced MRSI or Ernst‐angle MRSI has yet to be conducted.

The phantom data showed the feasibility of using low‐power ^31^P dual‐band pulses in multi‐echo approaches instead of high‐power demanding adiabatic pulses. In addition to increased SNR, T_2_ information of the metabolites can be estimated with this implementation. Understanding MR relaxation parameters such as T_2_ is necessary for absolute quantification of the metabolites. Furthermore, it can also provide insights into the molecular behavior of metabolites. Changes in T_2_ relaxation times, for instance, can be indicative of various pathological conditions.^29^, ^30^, ^31^ In the brain, van der Kemp et al^21^ reported T_2_ relaxation times for the metabolites GPC, PC, and PE at 7 T. They utilized composite block pulses with a refocusing bandwidth of 4 ppm, covering the frequency range encompassing PDE and PME. In our study, we observed shorter T_2_ values for these metabolites. This may be explained by the sensitivity of the dual‐band pulse to the B_0_ inhomogeneity where this main field inhomogeneities can lead to line broadening, distortion, or signal loss in MRS spectra. To gain insight into how B_0_ inhomogeneity affected the signal level of the desired metabolites, we simulated the effects of different offset frequencies of the dual‐band RF pulse on the signal intensities of PDE and PME metabolites. We observed a notable sensitivity of the pulse to the frequency offset. For instance, a 0.5 ppm (~60 Hz) shift in the RF pulse offset resulted in a substantial signal alteration. Analyzing the simulation results will help to understand how to mitigate or correct for B_0_ inhomogeneity in the actual MRS experiments to obtain more accurate metabolite quantification. Furthermore, considering the relatively long delta TE, we may not have maintained the Carr‐Purcell‐Meiboom‐Gill (CPMG) condition for all the chemical shifts, thereby resulting in distinct T_2_ values compared to the aforementioned literature. On the other hand, Several studies have reported the presence of background signals in the PME region such as mobile phospholipids and 2,3‐diphosphoglycerate (DPG), contributing to broadened baseline beneath PME resonances and additional complexity to the resonances downfield P_i_ toward PMEs.^32^, ^33^ The varying T_2_ values observed across different approaches (dual‐band, composite, and adiabatic) could be influenced by the different refocusing profiles employed, impacting signals potentially underlying the PME resonances.

Under poor B_0_ shimming conditions, yet negligible to the bandwidth of the RF pulses, the ability to detect the metabolites of interest is compromised due to shortened T_2_* and the resulting broadening of spectral lines. By employing multi‐echo spectroscopic imaging instead of FID CSI, we can improve SNR, regaining sensitivity in resolving the metabolites of interest. This approach can be particularly valuable in body organs characterized by substantial susceptibility differences among various tissue types, as opposed to the brain where effective B_0_ shimming is attainable. Beyond improving the SNR with T_2_‐weighted enhancements, multi‐echo MRSI permits T_2_ estimation for each metabolite. Deviation of the dual‐band RF pulse offset results in incomplete refocusing of the PME and PDE signals, thereby introducing a signal loss and inaccurate T_2_ values. While this decrease in signal levels will not impact the ratio between the PME and PDE signals, given that both groups will be equally affected, the low signal‐induced uncertainty may potentially affect pathological assessments reliant on the evaluation of the PME and PDE ratio. Nonetheless, low‐power multi‐echo spectroscopic imaging can benefit from mitigation strategies, such as the implementation of effective local B_0_ shimming techniques for enhancing magnetic field homogeneity, for a more accurate PME and PDE ratio determination. In addition to B_0_ inhomogeneities, body applications may face additional problems that can impair image quality. Patient movement, including respiratory and cardiac motion, and B_1_ inhomogeneity in a large field of view (often arising from surface coils) can affect the refocusing efficiency of the dual‐band pulse, leading to an inhomogeneous loss of signal. Using gating techniques, a whole body birdcage coil^22^ and/or B_1_ shimming can help mitigate the effects of motion and B_1_ inhomogeneities, respectively. By addressing these challenges, we may enhance the applicability of our dual‐band SLR RF pulse in body cancer research.

## 5 CONCLUSION

Our work introduced a dual‐band SLR RF pulse designed for ^31^P MRSI, specifically targeting metabolites of interest. While adiabatic pulses, with their high time–bandwidth (TBW) products, offer excellent selectivity, they also result in increased RF power deposition. This study presents an avenue for improving phosphorus imaging at 7 Tesla, achieving enhanced SNR and reduced SAR, with potential applications in body cancer research and clinical diagnostics.

## CONFLICT OF INTEREST STATEMENT

None of the authors have a conflict of interest to disclose.

## Supporting information


**Figure S1.** Simulated signals of GPC, GPE, PC, and PE, and modulated FA series. The simulated variable echo train is depicted in black. GPC, GPE, and PE signal is constant until 1260 ms (echo 84), the PC signal starts to decrease at ~900 ms. Echo‐spacing = 15 ms, TR = 5500 and ETL = 100.
**Figure S2**. (A) SNR of GPC as a function of number of echo pulses in multi‐echo MRSI with 180 pulses. The noise is taken into account with the same T2 decay weighting as the signal. (B) SNR as a function of SAR in multi‐echo MRSI compared with FSE with low FAs. SAR is proportional to the square of the flip angle. The maximum SNR of 1.76 was achieved for FSE with maintaining the signal for a relatively long time of 1260 ms. SNR values were calculated from Equation 1. For multi‐echo MRSI, the maximum SNR was 2.13 after taking T_1_ relaxation effect into account.

## Data Availability

The data that support the findings of this study are available from the corresponding author upon reasonable request.
