# Supplementary material for: 31P multi‐echo MRSI with low B1 + dual‐band refocusing RF pulses
Source: NMR Biomed. 2024 Oct 10;38(1):e5273. doi: 10.1002/nbm.5273 (PMC11602691; doi:10.1002/nbm.5273)
Supplement: Supplementary file 1 — Figure S1. Simulated signals of GPC, GPE, PC, and PE, and modulated FA series. The simulated variable echo train is depicted in black. GPC, GPE, and PE signal is constant until 1260 ms (echo 84), the PC signal starts to decrease at ~900 ms. Echo‐spacing = 15 ms, TR = 5500 and ETL = 100. Figure S2. (A) SNR of GPC as a function of number of echo pulses in multi‐echo MRSI with 180 pulses. The noise is taken into account with the same T2 decay weighting as the signal. (B) SNR as a function of SAR in multi‐echo MRSI compared with FSE with low FAs. SAR is proportional to the square of the flip angle. The maximum SNR of 1.76 was achieved for FSE with maintaining the signal for a relatively long time of 1260 ms. SNR values were calculated from Equation 1. For multi‐echo MRSI, the maximum SNR was 2.13 after taking T1 relaxation effect into account. [file NBM-38-e5273-s001.docx]

**Supplementary Materials**

We simulated the use of variable FAs FSE in the 31P regime as a new approach for gaining SNR per unit of time within specific absorption rate (SAR) limits. The simulation data present the effect of extending the echo train length (ETL) on the PDE and PME signals by using refocusing FA modulated FSE technique. T_1_ and T_2_ of the metabolites used in simulation are listed in Supplementary Figure 1A. TR=5.5s, nominal FA=15, maximum FA=130, and echo spacing=15ms. Weighted average ${SNR}_{wa}$ was calculated as in Eq. 1.

$S_{wa}=S0\frac{1+2\sum_{i=1,..,n} Siw_{i}}{1+2\sum w_{i}}{, SNR}_{wa}=\frac{S_{wa}}{\sigma_{wa}}= SNR0\frac{1+2\sum_{i=1,..,n} Siw_{i}}{\sqrt{1+2\sum w_{i}^{2}}}$ Eq.1

$$S_{wa}=S0\frac{1+2\sum_{i=1,..,n} e^{-2i\Delta TE/T_{2}}}{1+2\sum e^{-i\Delta TE/T_{2}}}$$

Eq.2

$${SNR}_{wa}=SNR0\sqrt{\left( 1+2\sum_{i=1}^{n} e^{-2i\Delta TE/T_{2}} \right)}$$

Where $S0$, $Si, SNR0$, $w_{i}$, $S_{wa}$and $\sigma_{wa}$ represent free induction decay (FID) signal, $i$^th^ echo signal, FID SNR, signal weight, weighted average signal and noise, respectively. The signal itself was used as signal weight. To compare, ${SNR}_{wa}$and SAR of a multi echo sequence with full refocusing 180-degree RF pulses with the same echo spacing of 15 ms and the same number of echo pulses (100) was calculated as previously described (20). FA squared was calculated as an alternative to SAR.

Figure 1 shows the simulated signals of PDE and PME by using the modulated FA FSE approach. It shows the flip angle train and resulting signal, which rapidly reaches pseudo-steady-state (PSS) conditions. Figure 2 shows SNR comparison for the GPC signal. The multi-echo method with a number of 20 full refocusing 180-degree RF pulses resulted in an SNR weighted average of 2.13 (considering T1 relaxation effect). FSE with modulated FAs gave an SNR weighted average of 1.76 at the same FA squared of 673.

Simulations show that we can achieve higher SNR with a full 180-degree multi-echo sequence than a modulated FAs FSE sequence. Therefore, Multi-echo ^31^P MRSI with full refocusing results in higher SNR at the same SAR level compared to the fast spin echo technique with modulated refocusing flip angles.

*
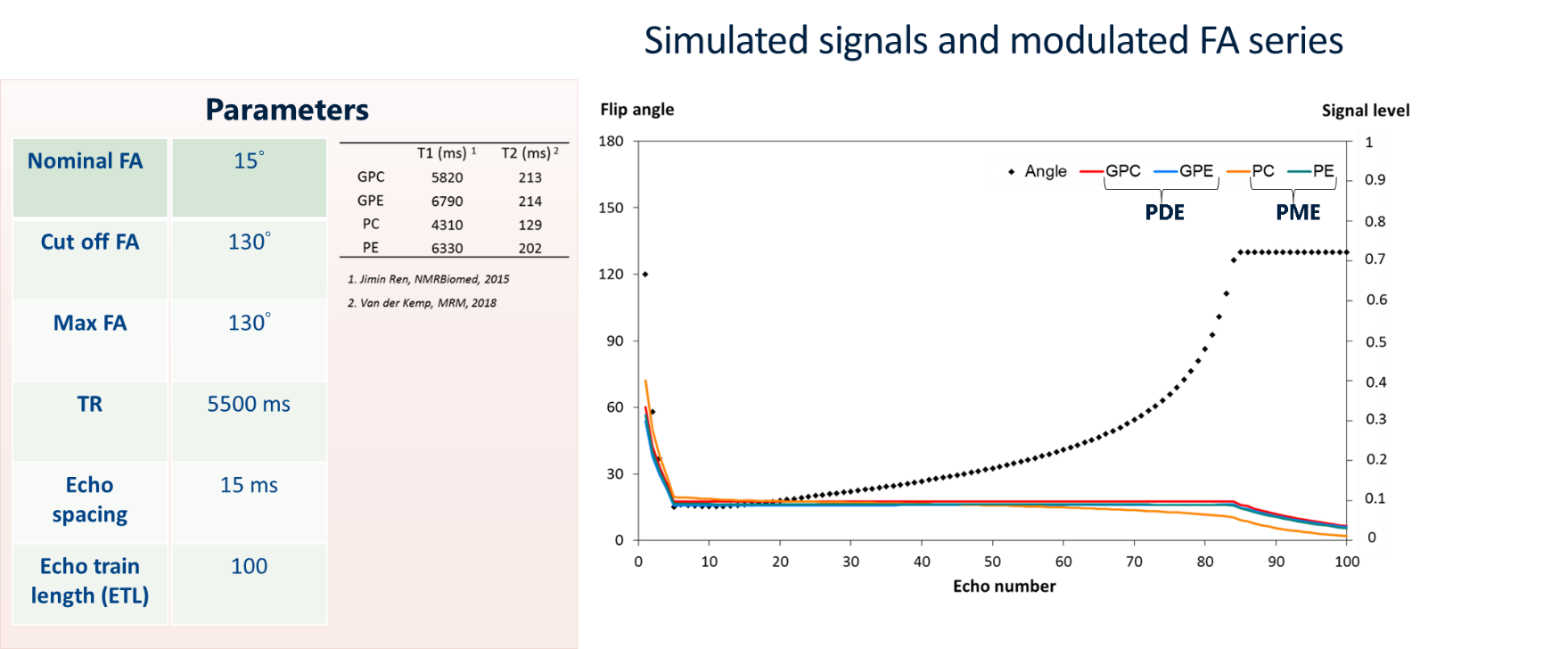
*

**Supplementary Figure 1**. Simulated signals of GPC,GPE, PC and PE, and modulated FA series. The simulated variable echo train is depicted in black. GPC, GPE and PE signal is constant until 1260 ms (echo 84), the PC signal starts to decrease at ~900 ms. Echo-spacing= 15 ms, TR = 5500 and ETL = 100.


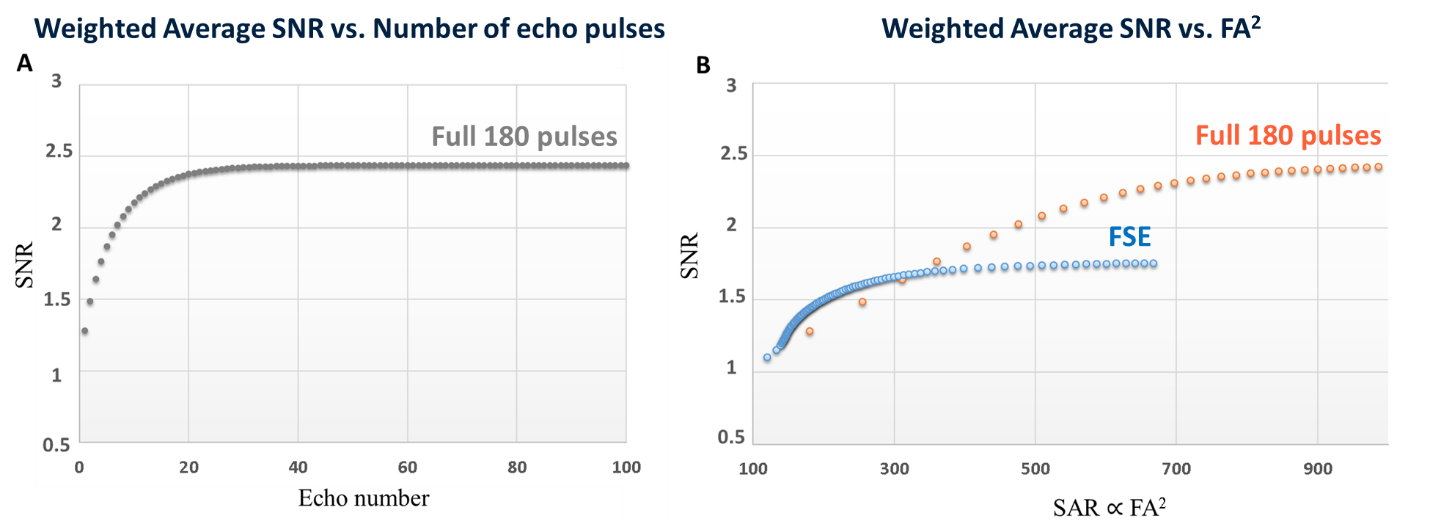


**Supplementary Figure 2**. (A) SNR of GPC as a function of number of echo pulses in multi-echo MRSI with 180 pulses. The noise is taken into account with the same T2 decay weighting as the signal. (B) SNR as a function of SAR in multi-echo MRSI compared with FSE with low FAs. SAR is proportional to the square of the flip angle. The maximum SNR of 1.76 was achieved for FSE with maintaining the signal for relatively long time of 1260 ms. SNR values were calculated from Equation 1. For multi-echo MRSI, the maximum SNR was 2.13 after taking T_1_ relaxation effect into account.
